# The progene hypothesis: the nucleoprotein world and how life began

**DOI:** 10.1186/s13062-015-0096-z

**Published:** 2015-11-26

**Authors:** Anatoly D. Altstein

**Affiliations:** Institute of Gene Biology RAS, NF Gamaleya Federal Center of Epidemiology and Microbiology, IM Sechenov First Moscow State Medical University, Moscow, Russia

**Keywords:** Origin of life, First genetic system, Progene, Origin of replication and translation, Genetic code, Selection of organic compounds

## Abstract

**Abstract:**

In this article, I review the results of studies on the origin of life distinct from the popular RNA world hypothesis. The alternate scenario postulates the origin of the first bimolecular genetic system (a polynucleotide gene and a polypeptide processive polymerase) with simultaneous replication and translation and includes the following key features:

1. The bimolecular genetic system emerges not from mononucleotides and monoamino acids, but from progenes, namely, trinucleotides aminoacylated on 3′–end by a non-random amino acid (NpNpNp ~ pX ~ Aa, where N—deoxyribo- or ribonucleoside, p—phosphate, X—a bifunctional agent, for example ribose, Aa—amino acid, ~ macroerge bond). Progenes are used as substrates for simultaneous synthesis of a polynucleotide and a polypeptide. Growth of the system is controlled by the growing polypeptide, and the bimolecular genetic system emerges as an extremely rare event. The first living being (virus-like organism protoviroid, *Protoviroidum primum*) arises and reproduces in prebiotic liposome-like structures using progenes. A population of protoviroids possessing the genetic system evolves in accordance with the Darwinian principle. Early evolution from protoviroid world to protocell world is shortly described.
2. The progene forming mechanism (NpNp + Np ~ pX ~ Aa) makes it possible to explain the emergence of the prebiotic physicochemical group genetic code, as well as the selection of organic compounds for the future genetic system from the racemic environment.
3. The protoviroid is reproduced on a progene basis via replicative transcription-translation (RTT, the first molecular genetic process) that is similar to its modern counterparts. Nothing is required for the emergence and reproduction of the protoviroid except for progenes and conditions for their formation.
4. The general scheme of early evolution is as follows: prebiotic world → protoviroid (nucleoprotein) world → protocell (DNA-RNA-protein) world → LUCA (Last Universal Common Ancestor) → modern cell world. This scheme exclude the existence of an independent RNA world as predecessor of the cellular world.

**Reviewers:**

Dr. Thomas Dandekar, Dr. Bojan Zagrovic and Dr. Anthony Poole

## Background and general approach

The first accepted scientific hypotheses explaining the origin of life were proposed by Oparin [1] and Haldane [2]. According to these hypotheses, the first living organisms were represented by multimolecular complexes that contained proteins, polycarbohydrates, and lipids. Compartmentalized and metabolically active, they had characteristics considered at the time as main attributes of life. Consistent with scientific views of the first half of the 20th century, it was presumed that such organisms were able to reproduce and evolve. The above hypotheses had stimulated an interest in that issue and prompted experiments in prebiotic chemistry started by Miller [3]. In the subsequent 60 years, there has been a multitude of impressive results proving the possibility of synthesis of a variety of organic matter, including amino acids, carbohydrates, fatty acids, nucleotides and aminoacyl nucleotides on the prebiotic Earth [4–10]. By the end of the 1960s, it became evident that a hypothesis that does not invoke genetic system as a necessary component of a living organism, cannot explain the origin of life and its evolution. Nevertheless, the Oparin-Haldane hypothesis retained its validity until 1980s [11] and was widely used in teaching biology.

In 1968, a hypothesis that it might have been RNA rather than proteins that constituted the original molecules of life [12, 13] was proposed. In 1983, an experimental model RNA-polymerase reaction that did not involve protein enzymes was developed in L. Orgel’s laboratory [14]. After ribozymes were discovered [15, 16], this idea became the new explanation of the origin of life: the RNA world hypothesis ([17–19], see also [20]) that replaced the Oparin-Haldane one. This idea has become the most accepted origin of life hypothesis and found its way into textbooks. According to this hypothesis, first organisms did not contain proteins but were constructed of RNA that served both as genetic material and the life processes supporting enzymes (replication, metabolism, synthesis of lipid membranes). The clearest conception of the first RNA genetic system was given by Cech [18]: it is a system containing two RNA molecules, a gene and a polynucleotide processive polymerase that is complеmentary to the gene. The RNA world hypothesis has since triggered research on ribozymes [21–28] to demonstrate various enzymatic activities, including ligases, distributive (but non-processive) RNA-polymerases, nucleases, aminoacyl tRNA synthetases and other types of activities by polynucleotides of up to 300 bases in length. Polydeoxyribonucleotides can also possess enzymatic activity [29]. The discovery of the ribosomal 28S RNA peptidyl transferase activity [30] lends a strong psychological support for the RNA world hypothesis. This discovery led to the assumption that the appearance of RNA preceded that of proteins in the first living organisms.

By early 1980s the data indicating the impossibility of a spontaneous synthesis of long polynucleotides in prebiotic conditions started to accumulate. A phenomenon of the stereochemical inhibition was discovered by Joyce et al. [31]: inclusion of an incorrect nucleotide (for example, the inclusion of an L-nucleotide into the D-chain) during the non-enzymatic template synthesis of a polynucleotide in a racemic environment, is, in fact, possible (the L-nucleotide must be in syn-conformation), but that would preclude further template inclusion of either D-, or L-nucleotides into the same chain.

Given that in a prebiotic environment a variety of nucleotide stereoisomers must be present, reproduction of polynucleotides would take place under the “replicative chaos” conditions that would preclude the possibility of synthesis of long chains and template replication [32, 33]. Recognition of this fact had led to an important correction to the RNA world concept. It was thought that life had most likely started not from the RNA world, but rather from a relatively undefined pre-RNA one, i.e. the first organisms’ genetic system was based on nucleotides which contained, instead of chiral ribose, a different achiral compound diminishing the threat of a “replicative chaos” [34–36]. At any rate, the RNA world succeeds the pre-RNA one; later, in the process of evolution, organisms appear that use DNA as genetic material and synthesize proteins, which complement and replace ribozymes [37, 38]. A problem of arising and evolution of genetic code and translation mechanism has appeared and as of yet does not have a definite solution (see [39–43]).

Alternatively it is possible that the origin of life from the very start involved nucleic acids and proteins [44–48], and hence a concept of a nucleoprotein world is suggested. In 1977 Eigen and Schuster proposed a theoretical system for synthesis of peptides and short polynucleotides—«The Hypercycle Theory» [44]. They introduced an important concept named «catastrophe of errors» and “threshold of errors” that proceeds due to inaccuracy of enzymeless template directed polynucleotide synthesis as a result of G pairing not only with C (three H-bonds) but also with U (two H-bonds). This leads to destruction of genetic information. Any new hypothesis of origin of a genetic information has to take this phenomenon into account. «Hypercycle» (a system of short interdependent polynucleotides translating oligopeptides) was proposed for overcoming «catastrophe of errors». A realistic mechanism of the origin of the system (especially translation) was not considered. Such a mechnism for simultaneous synthesis of a polynucleotide and a polypeptide was proposed by Altstein and Kaverin in 1980 [48], then Altstein in 1987 [49] and in update form in this paper. The major part of this concept (the progene hypothesis) was published mainly in Russian or in short form [48–54].

The terms “life, living organism” can be defined in different ways [55]; no universally accepted definition exists. Definition suggested by NASA (“Life is a self-sustained chemical system capable of undergoing Darwinian evolution”) seems to be quite convincing as it emphasizes two main attributes of life: the ability to reproduce and evolve. I use the following definition which fully reflects the spirit of the NASA formula, while especially emphasizing the role played by carbon containing polymers and template processes: “Living organism is an open integral system which consists of carbon containing polymers and reproduces and evolves on the basis of template directed processes”.

The genetic system responsible for an organism’s reproduction, as well as for fixation of the advancements in its evolution is an imperative component of a living being. Contemporary genetic systems are based on polynucleotides and the template principle of their reproduction [56]. Our hypothesis is premised on the idea that the first living organism has to possess a principally similar genetic system. The origin of life is the origin of a gene [57]. Therefore, any biolike structures (such as Oparin’s coacervates, Fox’s microspheres, liposomes, fatty acid vesicles and others) that do not possess a genetic system of their own are not to be considered as living organisms.

What should the nature of a living organism be like? Many researchers agree that the first living being must be of the cell nature (protocell), as life is impossible without compartmentalization and metabolism. However, the genetic system of such a cell from the very beginning has to include several genes that control the reproduction of genome, cell membrane formation, and metabolic chains. In prebiotic conditions, genes can only form as a result of nucleotide combinatorics that very seldom realize into a necessary function. It is logical to presume the emergence of a single (the first) self-reproducing gene to be the origin of the first genetic system. A living protocell, which requires several genes, is too complex to become the first organism. It evolves from a monogenic non-cellular organism. The first genetic system itself is *de facto* the first organism. However, this organism cannot do without compartmentalization and metabolism either. Our approach is based on the idea that the prebiotic environment provides for abiotic metabolism and compartmentalization. The extensive literature exists on synthesis of various organic substances, important for emergence of life (amino acids, fatty acids, carbohydrates, nitrogen bases, nucleosides, and nucleotides) in conditions that are similar to prebiotic ones. It’s especially necessary take into consideration a very important research of Sutherland and coworkers on simultaneous synthesis of pyrimidine nucleotides and other compounds, necessary for the origin of life, in approximately prebiotic conditions [6–8]. Activation of organic substances is possible, as well as formation of peptide bonds between amino acids, and phosphodiester (PDE) bonds between nucleotides. Abundant formation of lipid structures that would allow for compartmentalization of first living organisms is thought to be possible [58–65]. This problem has been discussed in details in Deamer’s book [66].

If one accepts the premise that abiotic metabolism and compartmentalization take place in prebiotic conditions, the main question to be posed would be one of the mechanism of formation of the first gene, and therefore, of the first non-cellular organism. Such organism must consist of two mutually dependent components: 1) gene-polynucleotide; 2) processive polymerase. Subsequently, the gene encodes itself, as well as the polymerase, the latter reproduces the gene and itself. The system in question needs to be bimolecular for the successive Darwinian evolution to become possible.

The choice of the chemical nature of the polymerase is exceptionally important. If the polymerase happens to be a ribozyme [18], the problem can be solved within the RNA world hypothesis which is challenged above. It is very unlikely that a short (<200 base) ribozyme complementary to its own template can act as an efficient processive polymerase. Long polynucleotides cannot be synthesized due to the “replicative chaos” problem, as well as to the inefficiency of non-enzymatic synthesis of biopolymers. If the polymerase is of protein nature, a fundamentally different concept is needed that would explain the origin of the genetic code and translation. Such a concept (the progene hypothesis) is presented earlier [49] and as updated one in this paper. The main goal of this hypothesis is to suggest a single mechanism that would explain: 1) emergencе and reproduction of a bimolecular genetic system consisting of a polynucleotide gene and a protein processive polymerase encoded in that gene; 2) mechanism of selection of constituents of genetic system, including arising of homochirality; 3) arising of the genetic code and translation. The nature, the way of existence, and the evolution of the first living organism (protoviroid) will be briefly discussed.

Testing of some of the elements of this hypothesis involving stereochemical analysis and chemical experiments is principally possible.

## The progene hypothesis

According to the existing beliefs, prebiotic polynucleotides were synthesized from mononucleotides, and polypeptides from monoamino acids. It is this particular idea that prevents one from understanding neither the principles of compounds selection for the growing future biopolymer, nor the connection between nucleotides and amino acids (Aa), i.e. the emergence of the genetic code. In accordance with the progene hypothesis, nucleotides first unite into amino acylated trinucleotides (progenes), which further become the sole substrate for the simultaneous synthesis of the polynucleotide and polypeptide, while the sequence of amino acids gets encoded in the polynucleotides, and the emerging polypeptide possesses properties of the processive polymerase (the progene ligase). The hypothesis is presented as the central postulate and its three consequences.

### The central postulate: progene forming mechanism

**Fig. 1:**
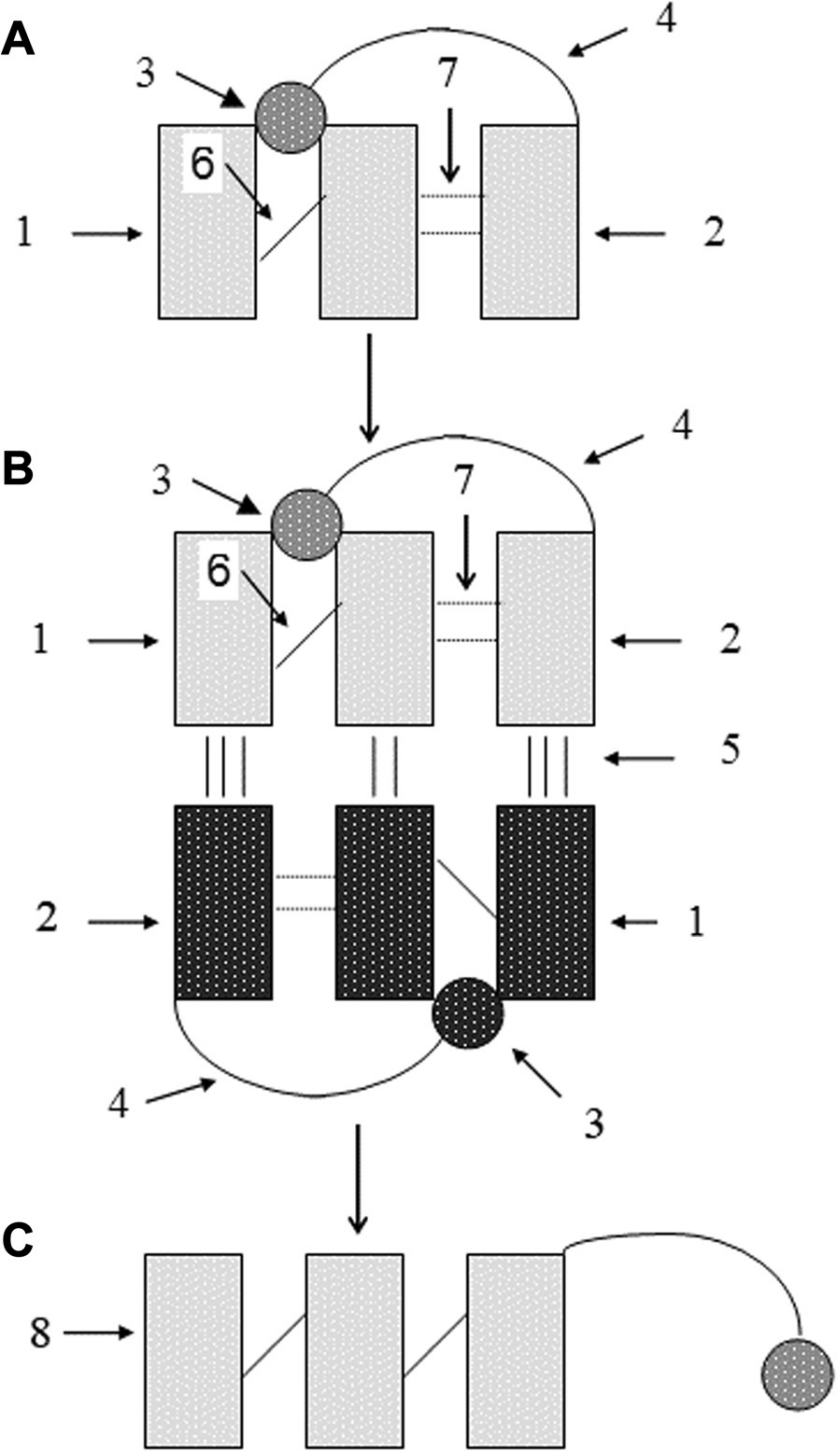
The mechanism of the progene formation. 1—dinucleotide; 2—aminoacyl nucleotide; 3—amino acid; 4–3′ “tail”(p ~ pX ~ Aa, see text); 5—complementary H-bonds between “triplets”; 6—phosphodiesther bond; 7—stacking between nucleotides; 8—progene. **a** Formation of a unstable “triplet” between a dinucleotide (DN) and a aminoacyl nucleotide (AAN) due to stacking and specific interaction between the amino acid (Aa) and DN. **b** Formation of complementary interaction between two unstable “triplets”; the condition for formation of the template-directed phosphodiesther bond (PDEB) takes place between 2nd and 3d (amino acyl) nucleotides. **c** The progene; arises on B-stage after PDEB formation between the DN and the AAN; contains the nucleotide triplet, Aa specific for the DN and two macroerges (NpNpNp ~ pX ~ Aa)

Progenes (5′NpNpN3′p ~ pX ~ Aa, where N—deoxyribo- or ribonucleoside, p—phosphate, X—a bifunctional agent, for example, ribose, Aa—amino acid, ~ macroerge bond) are formed from two components: a dinucleotide (DN, 5′NpNp) and an special aminoacyl nucleotide (AAN, 5′Np ~ pX ~ Aa) in three stages (Fig. 1). At the first stage DN and AAN physically join into an unstable (imperfect) “triplet” via stacking interaction between AAN and the 2^nd^ nucleotide of DN, as well as via specific interaction of amino acid with DN (Fig. 1a). The lifetime of such “triplet” depends on stacking energy and interaction between the amino acid and DN. If the lifetime of this “triplet” (~10^−8^–10^−9^s) is greater or equal to the time needed for overlapping by another complementary unstable “triplet”, a complementary complex consisting of two “triplets” is formed at the 2^nd^ stage, and lifetime of the two unstable “triplets” increases drastically (up to 10^−3^–10^−5^s, Fig. 1b). Formation of the complementary complex depends critically on the energy of the interaction between the amino acid and DN at the first stage, on stacking between the adjacent nucleotides, and on the number of hydrogen bonds between complementary nucleotides at the 2^nd^ stage. The complementary interaction, achieved at the 2^nd^ stage, increases the probability of phosphodiesther (PDE) bond formation between the 2^nd^ nucleotide of DN and AAN. Activation of the 3′hydroxyl substantially increases the probability of the PDЕ bond formation in comparison with 5′ hydroxyl activation [67, 68]. As a result, a progene is formed—a trinucleotide amino acylated by a non-random amino acid on 3′–“tail” (NpNpNp ~ pX ~ Aa) (Fig. 1c). A convincing demonstration of the role of a template directed mechanisms for PDE bond formation has been given by Orgel and his colleagues [14, 69–71] and has since become generally accepted. The nature of catalysts for PDE bond formation between DN and AAN is unclear. It is possible participation of a metal ions (Mg^2+^, Zn^2+^, Fe^2+^ or others), connected with AAN α-phosphate, in this process.

Not only imperfect but also normal short (tri- and longer) oligonucleotides could serve as templates for a synthesis of progenes. It is more convenient for experimental testing of the hypothesis.

This postulate shows how the interaction between amino acids and dinucleotides, as well as the one between nucleotides, create conditions for joining of a specific amino acid to a trinucleotide. The idea that the amino acid helps nucleotides to join by keeping them together was first presented earlier [48, 72]. A possibility of the specific interaction of amino acids with nucleotides has been discussed by many researchers, but is still considered unproved.

### Consequence 1. Emergence of the first genetic system from progenes and its self-reproduction on basis of the progenes

**Fig. 2:**
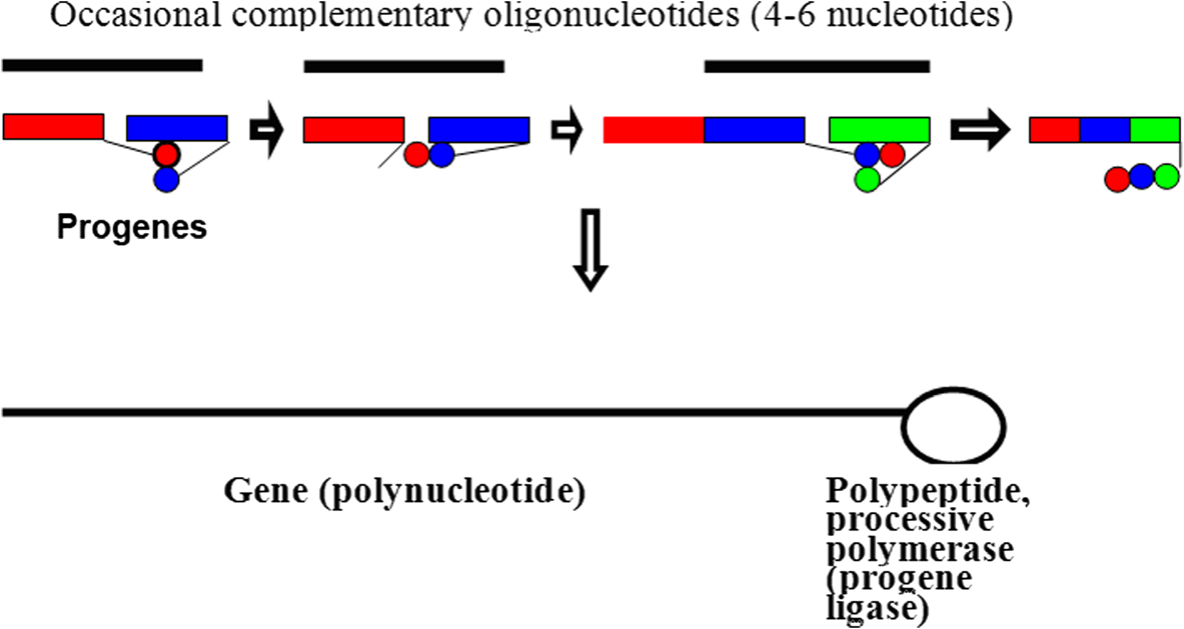
Arising of the bimolecular genetic system from progenes

Progenes are the substrate for the simultaneous formation of a polynucleotide and a polypeptide (Fig. 2). Two progenes approach each other via stacking and retaining of the 2^nd^ progene by the amino acid of the 1^st^ one, and are stabilized by complementary oligonucleotide consisting of 4–6 nucleotides. The N-end of the amino acid of the 2^nd^ progene approaches the activated C-end of the 1^st^ progene’s amino acid; a dipeptide connected with the 2^nd^ progene is formed. The N-ended amino acid of the dipeptide (Glu or Asp) (due to of their acid side groups) is able to affect the formation of PDE bond between the 1^st^ and the 2^nd^ progenes (the basic catalysis). As a result, hexanucleotide is formed, which is connected to a dipeptide. The dipeptide possesses an increased ability to retain the next progene, and the entire process repeats, as the dipeptide gets transferred onto the 3^rd^ progene (transpeptidation) and helps the latter join the hexanucleotide. The nonanucleotide with the tripeptide is formed. Thus, a “genotype (the order of triplets in the polynucleotide)—phenotype (the order of Aa in the polypeptide)” connection is formed because every triplet “memorizes” the Aa encoded by it. Arising of “genotype—phenotype” connection one of the most complicated problems of the origin of life.

Growth of the system occurs with the involvement of templates (short prebiotic oligonucleotides) that increase probability of PDE bond formation. The growing peptide constantly participates in the formation of the system: if the latest Aa to join the peptide increases the probability of next progene inclusion, growth of the whole system continues; if the latest Aa diminishes the probability of the next progene’s inclusion, the growth of the system stops. Hence the enzyme, directing template processive synthesis of the polynucleotide and itself, is formed simultaneously with its gene. It could say the enzyme grows in close interaction with its future substrate. As extremely rare event, emerging of such a pair (the gene and enzyme) is the result of a huge number of attempts during tens and hundreds million year period. The appearance of a linked pair “gene—proсessive polymerase (progene ligase)” signifies the emergence of the first living being from the progenes due to chemical (non—Darvinian) evolution.

One must bear in mind that according to the progene hypothesis, the process of the first living being’s formation is a chemical reaction (polymerization of progenes) with an extremely low output of the final product (~10^−35^, if we consider the combinatorics of amino acids and inclusion of incorrect structures at early stages of system’s growth).

Proposed scheme of emergence of genetic system allows for the chirality problem’s solution. The progenes are mostly chirally pure, D- or L (see Consequence 2. Chemical nature of progene and constituent selection). A probability of the L-progenes’ inclusion into the chain built of D-nucleotides on the D-template is negligible: in accordance with the rule of stereochemical inhibition [31, 32], it is only the first nucleotide of L-progene, being in the syn-conformation, that forms complementary connection with the template; the 2^nd^ and the 3^rd^ nucleotides do not form such connection. Therefore, retention of an “incorrect” progene next to a “correct” progene on the “correct” template will be much weaker. The responsibility to completely exclude “incorrect” progenes lies with the polymerase that is supposed to acquire substantial stereospecificity in the process of its formation.

Formation of the living world based on D–nucleotides and L–amino acids would a result of fixation of one of two possibilities (see also Consequence 2. Chemical nature of progene and constituent selection). Genetic system that arises from progenes will reproduce based on them in the same conditions. It is important that the processive polymerase (progene ligase) is able: 1) to have tropism towards the 3′end of the polynucleotide template; 2) to retain near template two progenes complementary to it (or the end of the growing chain and the successive progene) and to use progenes as the only substrate; 3) to promote first the formation of the peptide bond between the progene’s amino acid and growing polypeptide, and then PDE bond between the progene and growing polynucleotide; 4) to move from the 3′to the 5′template end up to the 3′end of the growing chain; and 5) having reached the 5′template end, to remain connected to the 3′end of the new chain. The polymerase needs to have a diameter of 15–20 Å, which corresponds to a globular polypeptide consisting of 80–120 Aa’s.

**Fig. 3:**
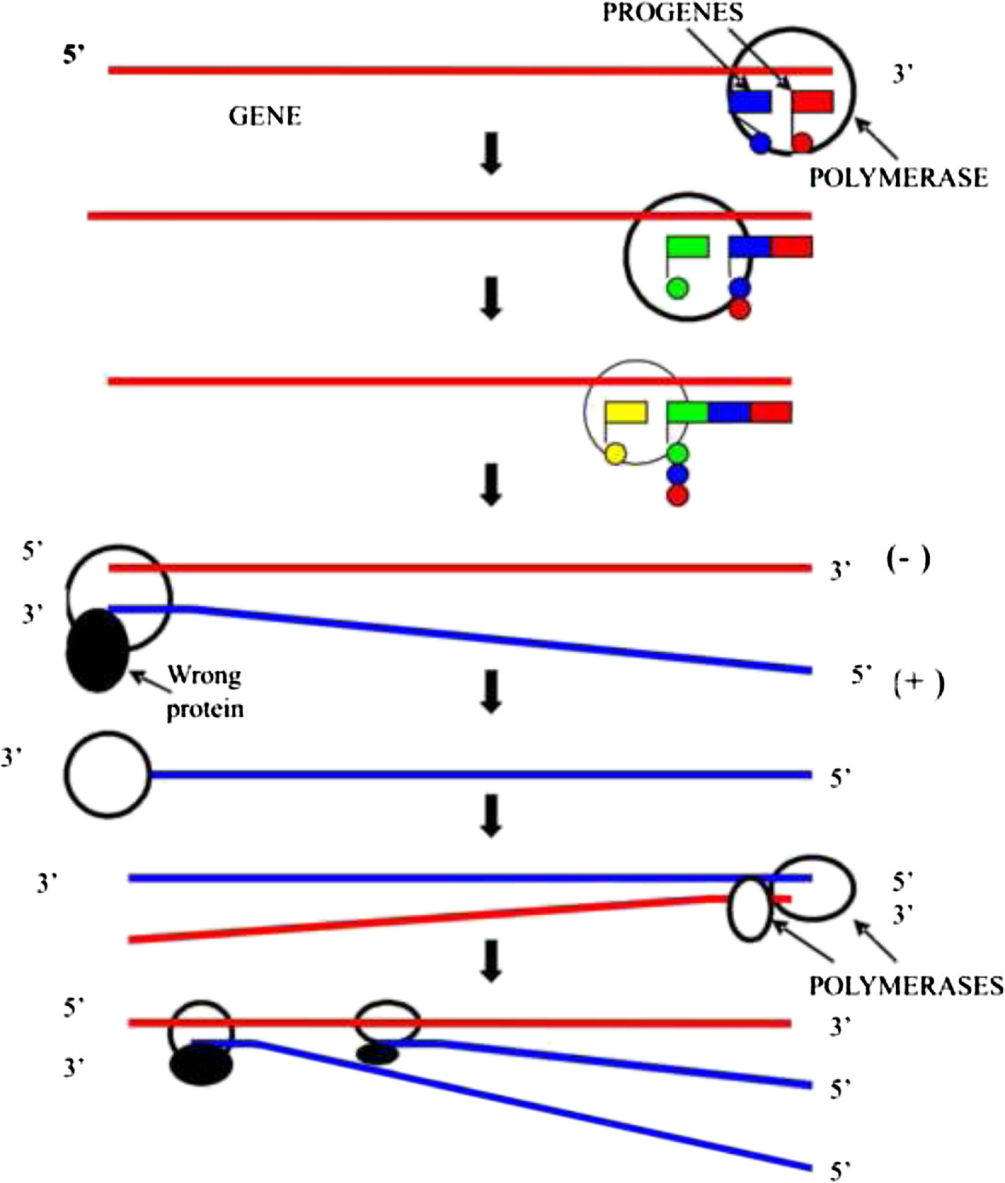
Simultaneous replication, transcription, and translation (RTT process) of the bimolecular genetic system based on the progenies.

The principle of self-reproduction (simultaneous replication, transcription, and translation) of the genetic system based on the progenes is shown on the scheme (Fig. 3). The polymerase located on the 3′end of the gene moves to its 5′end, connecting the progenes complementary to the template. Simultaneously, synthesis of the complementary polynucleotide occurs via replication and transcription, since the original gene is represented by a (−) strand, as well as synthesis of an “incorrect” protein (it is minus strand translation). The polymerase moves to the 3′ end of the newly synthesized (+) strand and replicates it while simultaneously forming a new polymerase molecule and a new (−) strand. Two polymerase molecules further create two new (+) strands. Then two new (−) strands and two additional molecules of the polymerase (totally 4 polymerase molecules) will be synthesized (not shown on the scheme). Thus, the first molecular genetic process is presented by conjugated replication, transcription, and translation (the RTT process performed by one enzyme). The RTT principles are close to today’s template directed separated processes.

Apparently, the bimolecular genetic system is able to overcome the “catastrophe of errors” [44] using the enzymatic template polymerization of the progenes instead of template polymerization of mononucleotides without enzymes.

### Consequence 2. Chemical nature of progene and constituent selection

The proposed mechanism of progene formation allows us to solve, in a general form, the problem of constituent selection for the future genetic system. A trinucleotide is a minimal weak template, and formation of a complementary pair of imperfect “triplets” (Fig. 1) can only occur if all available components are optimal for this interaction. Stacking between adjacent nucleotides and the number of hydrogen bonds between complementary ones are important, as well as the ability to interact with amino acids that are candidates for the inclusion into the progene. Today’s nucleotides G, C, A, T, U that are based on pentoses, appear to be the most suitable nucleotides for the formation of the complementary structure consisting of two imperfect “triplets”.

Investigations of Orgel and his coworkers [14, 68–71, 73] has shown that template drastically stimulates formation of the PDE bond between nucleotides in non-enzymatic polymerase reactions; stacking between pyrimidines is insufficient for PDE bond formation, whereas stacking between purines is beneficial for this purpose. Stacking between a purine and a pyrimidine occupies intermediate position and allows for PDE bond formation that directed by template. Stability of the complementary complex that depends upon both stacking and number of hydrogen bonds between complementary nucleotides, affects PDE bond formation. That is why the optimal situation for the progene formation would be alternation of purines and pyrimidines that would provide sufficient stacking and number of hydrogen bonds between triplets not less than 8, i.e. “strong” nucleotides G and C (three H-bonds) dominate in triplets. Based on these principles (alternating purines and pyrimidines; prevalence of G and C), the following triplets are likely to dominate in progenes: GTG, ACG, GCA, GCG, CAC, T/UGC, CGT/U, CGC. T in the 2^nd^ position has advantages over U, as its 5–СН3 group is important for interaction with hydrophobic amino acids (see Consequence 3. The molecular mechanism of genetic code arising).

It is possible that no less than nine H-bonds are necessary between two imperfect “triplets” for progene formation. It could to assume that the 2,6–diaminopurine (2–aminoadenine) (A*) was used instead of A. A* differs from A only by an additional NH_2_ –group in 2–position of the nitrogen base and forms 3 H-bonds with T and U (T/U–A* pair is as “strong”as G-C). In this case 16 triplets (but not 8) could dominate in the progenes): GTG, GTA*, A*TG, A*TA* (T-group); GCG, GCA*, A*CG, A*CA (C-group); CA*C, CA*T, TA*C, TA*T (A-group); CGC, CGT, TGC, TGT (G-group) (U instead of T is possible in the first and third positions of triplets). Availability of the diaminopurine in prebiotic conditions is unclear but possible [74]. The diaminopurine was detected as an adenine substituting in S-2 L cyanophage DNA [75].

The majority of progenes have to be chirally pure on nucleotides (DDD or LLL). The dinucleotides LD and DL are not suitable due to stereoinhibition phenomenon [31]; DDL or LLD are not completely excluded, but are less probable than DDD or LLL. There are reasons to suggest that in progenes L-amino acids better correspond to D- than to L-nucleotides, and vice versa. This question requires further investigation. There are data on stereospecific influence of L-amino acids on synthesis of D-sugars [76, 77]. The chirality problem is solved finally in the process of arising bimolecular genetic system (see Consequence 1. Emergence of the first genetic system from progenes and its self-reproduction on basis of the progenes).

The most important moment in the mechanism of progene formation is creation of PDE bond between 2^nd^ and 3^rd^ nucleotides of the progene. It is known, that for the formation of PDE bond in non-enzymatic reactions, activation of 3′ hydroxyl of nucleotide is significantly more effective than activation of its 5′hydroxyl [67, 68]. Therefore, in progene, activated phosphate is in the 3′position. However, in ribose, activation of 3′end phosphate leads to formation of the 3′–2′ cyclophosphate that is not compatible with progene structure. This results in selection preferably of deoxyribonucleotides but not ribonucleotides into progenes. Hence, progenes have preferentially deoxyribonucleotide (or mixed) nature, and the genetic system that arises from them is based on DNA (or mixed nucleic acid), an idea that contradicts the accepted RNA world hypothesis. The obligatory T presence in GTG progenes explains why this nucleotide that is a part of today’s DNA: it has been the case from the very beginning. It should be noted that now is absent reliable data on prebiotic synthesis of deoxyribonucleotides but they can appear as result of future investigations.

One of the most essential components in progene formation is amino acyl nucleotide that becomes the 3^rd^ nucleotide of the progene. In our early papers [48–54] we supposed that AAN is a mixed anhydride between carboxyl group of amino acid and the 3′–β- (Np ~ p ~ Aa) [48] or γ-phosphate of nucleotide (Np ~ p ~ p ~ Aa) [49]. To achieve fundamental goal of the hypothesis (the simultaneous synthesis of polynucleotide and polypeptide chains from progenes) it is essential that 1) the 3^rd^ progene nucleotide has macroerge bond between α– and β–phosphates for the PDE bond with another progene to form; 2) an amino acid be linked to progene by macroerge bond for connecting with the amino acid of another progene; and 3) the length of the 3′-end amino acylated “tail” of the 3^rd^ progene nucleotide be sufficient for the interaction between Aa and first two progene nucleotides (see Fig. 1a and b). The triphosphate “tail” satisfies these requirements. But this structure of the “tail” becomes the source of major difficulties in experimental investigation of the mechanism of progene formation, due to its high lability caused by the following: 1) hydrolysis of the bond between carboxyl group of amino acid and the 3′–γ-phosphate of nucleotide; 2) possibility of intramolecular formation of cyclophosphate between the activated 3′–γ- and 3′–α–phosphates.

Therefore, the structure of amino acyl nucleotide (Nppp-Aa) proposed earlier needs to be changed to more realistic Np ~ pX ~ Aa (i.e. γ-phosphate is replaced by a bifunctional agent, and highly macroerge anhydride bond p ~ Aa by energetically weaker but more stable ether bond -O ~ Aa). It is possible that different bifunctional agents (X) such as ribose, ethylen glycol, aminoethanol, glycerol and others will become suitable. The probability of formation of such aminoacyl nucleotides in prebiotic conditions is not clear and have to be explored.

### Consequence 3. The molecular mechanism of genetic code arising

The mechanism of progene formation presumes specificity of the interaction between Aa linked to the 3^rd^ nucleotide, and DN (Fig. 1). At the same time, Aa contributes to progene formation and becomes included in its composition. Physicochemical nature of emerging genetic code was first described by Woese [78] who analyzed amino acid positioning in the genetic code table, but the essence of the specificity of the interaction between amino acids and nucleotides which leads to encoding is still unknown or even denied [12]. Now there are many publications that support a physicochemical nature of the genetic code arising (see it in more details in [79, 77–83]).

The progene hypothesis suggests a new possibility for solving this complex problem. Progene triplets correspond to today’s codons while progenes themselves are tRNA analogs serving as anticodons in the primary living world. If it is true that genetic code appeared in accordance with progene hypothesis, it can be expected that stereochemical analysis will reveal some similarity between the original physicochemical code and today’s one. Preliminary analysis has been conducted with the help of CPK models [50], as well as the Hyperchem computer software (54, Khairetdinov, Altstein, unpublished).

In accordance with the progene hypothesis, the rules for the analysis were as follows: 1) the trinucleotide was taken in the B-form; 2) the amino acid was fixed at its C-end on the 3′–γ-phospate of the 3^rd^ nucleotide; and 3) the N-end of any amino acid was linked with the oxygen atom of internucleotide phosphate (between the first and the second nucleotides), directed into the “major groove”, thus forming ionic or hydrogen bond (this being a standard interaction, uniform for all Aa). It turned out that in such interaction, the side groups of Aa are directed toward the nitrogen base of the middle (2^nd^) nucleotide and can interact with the latter (hydrogen bonds, hydrophobic interaction, Van-der-Waals contacts, dehydration of acceptors and donors of the hydrogen bonds). The field of interaction between Aa and DN is limited due to the length of amino acylated “tail” and allows one to see for all possible atomic contacts between Aa and DN to be revealed.

The main goal of the research is finding the optimal interactions of various amino acids with various DN and comparing the results with today’s genetic code.

Conducted research has shown that specificity is mostly determined by the 2^nd^ progene nucleotide. Computer-generated models of four dinucleotides (ТТ, СС, АА, GG) are presented in the addendum.

1. ТТ, NT (N is any nucleotide) owing to its 5–СН3 groups, has extensive area suitable for optimal hydrophobic interaction with canonic (Val, Leu, Ile, Phe, Met, Tyr, Trp) and non-canonic (n-Val, n-Leu, α-But) hydrophobic Aa. Carboxyl and hydroxyl groups of side radicals of dicarbonic and hydroxy Aa’s do not have sufficient opportunity for interaction with this nucleotide. This explains why T in 2^nd^ position encodes hydrophobic amino acids. U has not the 5–СН3 and therefore less suitable for hydrophobic interaction with Aa’s.
2. СС, NC is able to interact with all but basic Aa groups, including the most widespread small Aa (Аla, Gly) and also dicarbonic and hydroxy Aa’s. Hydrophobic Aa are their main competitors, but their hydrophobic interaction here is weaker than with TT. Hence C is suitable for encoding a small polar and non-polar Aa’s.
3. АА, NA, NA* is not convenient for hydrophobic interaction due to dehydration of the N7 atom. It is not convenient for the hydroxyl Aa, either: hydroxyl of Ser dehydrates N7 when it links with 6–NH of adenine as the H-bond acceptor, while linking with N7 as the H-bond donor, it dehydrates 6–NH group. Adenine is advantageous for dicarbonic Aa, especially for Glu (formation of H- bond between the radical’s carboxyl group and 6–NH of adenine without dehydration N7). This explains why A encodes dicarbonic Aa (and in the future their amines, as well). An interaction between DN CA and ANN Cppp-Glu is shown in the addendum (see GLU-CAC).
4. GG, NG is not convenient for hydrophobic interaction with Aa due to dehydration N7 and does not possess H-bond donors for the interaction with dicarbonic Aa, but is advantageous for Ser (H-bond with N7 without dehydration of 6–O). This explains rather strange positioning of Ser in the column of the genetic code table, which contains 2^nd^ nucleotide G. G is very convenient for Arg (two H-bonds with H-acceptors 6-O and N7) and for other basic Aa (Lys, His).

Data has been obtained indicating good principal correspondence between our stereochemical analysis for 13 of the 20 canonic Аа (i.e. Gly, Ala, Val, Leu, Ile, Pro, Phe, Met, Ser, Thr, Asp, Glu, Arg) with today’s genetic code [50]. Of these 13 Aa, 8 belong to the ones most abundant in prebiotic conditions: Gly, Ala, Val, Asp, Glu, Ser, Leu, Ile [84, 85]. The other 12 Аа appear to have been scantily represented in such conditions; of these 5 (Phe, Met, Pro, Thr, Arg) correspond with the today’s genetic code, four (Tyr, Trp, His, Lys) do not correspond, while three (Cys, Asn, Gln) give uncertain results in our stereochemical analysis.

Development of prebiotic physicochemical code table (Table 1) similar to the modern one becomes less difficult given that there are only 8 abundant progene triplets (or 16 if A* will be instead of A), and not 64 (see the Consequence 2. Chemical nature of progene and constituent selection) and only 8 abundant canonic and 3–4 non-canonic (аBut, n-Val, n-Leu) amino acids (4 groups), and not 20. It is important that during appearance of the prebiotic (primary) genetic code basic, sulfur-containing, aromatic Aa and amines of dicarbonic Аа are in negligable amounts or absent.

**Table 1:** Prebiotic physicochemical group genetic code^a^

2^nd^ 2^nd^ nucleotide	T	C	A	G
Codons	GTG	GCG	CAC	CGC
		ACG		TGC
		GCA		CGT
Amino acids	Hydrophobic nonpolar	Small polar and nonpolar	Dicarbonic	Hydroxy
	Val, n-Val, Leu, n-Leu, Ile, a-But	Ala, Gly [Ser, Asp]	Asp, Glu	Ser [Thr]

^a^If diaminopurine nucleotide (A*) was used for the progene formation instead of adenine nucleotide (A), four codons will be in each column (see Consequences 2 and 3): PuTPu, PuCPu, PyA*Py, PyGPy where Pu—purine (A* or G), Py—pyrimidine (T, U or C)

Features of the modern genetic code explainable by the progene hypothesis are as follow: 1) tripletness the progenes are triplets); 2) degeneracy (because the first and the third nucleotides of the progene are less specific than the second one; 3) non-overlapping and absence of commas (due to the mechanism of polynucleotide formation from the progenes, Fig. 2); 4) greater specificity of the first duplet and special role of the second nucleotide (see the mechanism of progene formation, Fig. 1, and the text of this section); 5) coding of the polar Aa’s by the purines in the second position of the codons (11 of 12) and of nonpolar Aa by the pyrimidines (7 of 9) (see the text of this section); 6) respective coding of Т for hydrophobic Аа, С for small polar and nonpolar Аа, А for dicarbonic Аа, and G for Ser and Arg (see the text of this section).

### Conditions of the emergence and evolution of the first self-reproducing progene-based genetic system (a short general scenario)

There are many hypotheses on chemical evolution of metabolism and compartmentalization in prebiotic period. The earliest Oparin-Haldane’s hypothesis of chemical and biochemical evolution proposed an idea of the primary “soup” containing of organic substances transforming step by step into multimolecular complexes and then into protocells (protobionts) [1, 2]. Many researches were done in prebiotic chemistry after Miller’s work [3] on the basis of this hypothesis [4–10, 66, 86–88]. It’s especially necessary take into consideration a very important research of Sutherland and coworkers on simultaneous synthesis of pyrimidine nucleotides and other compounds, necessary for the origin of life, in approximately prebiotic conditions [6–8, 88]. Important role of volcanic activity and meteorites in prebiotic syntheses is broadly recognized [5, 9, 10, 74, 89].

Some hypotheses attach significance to inorganic compartments in evolution of primordial chemistry and biochemistry for the origin of life. Wachtershauser proposed a hypothesis of surface metabolism on emergence of life at volcanic regions on surfaces rich in ions of heavy metals (iron, nickel and others) that were catalytically active and promoted CO_2_ fixation, leading to the growth of organic superstructures including synthesis of peptides [90–92]. Arising of biologic cellular organization and genetic mechanisms is, under this scenario, the result of prebiotic chemical evolution. Interesting opportunities was opened by Baross and Hoffman’s hypothesis on role of hydrothermal submarine vents in the origin and evolution of life [93]. According to Russell and Hall [94] the hydrothermal vents give rise to continuous flow reactors with thermal gradient that generate mounds of precipitate silica, clays, carbonates, iron-nickel sulfids and has inorganic cellular stucture (networks of inorganic compartments). These compartments appear to be a perfect environment for diverse organic syntheses [95], a good cradle for origin of a primitive life.

At present time there are three types of main compartments for primitive life described in scientific literature: 1) lipid vesicles (liposome-like and fatty acid membrane structure) [58–66, 89, 96]; 2) different inorganic compartments [91, 92, 94, 95, 97]; 3) Fox’s proteinoid microspheres [5]. Combined compartments might exist, for example, lipid vesicles inside inorganic compartments or inorganic particles covered with lipid membranes [89].

Based on the already known data and ideas, conditions of progene formation can only be described as an approximate scenario rather than a clearly defined hypothesis. It is presumed that conditions for abiotic synthesis and accumulation of organic matter necessary for emergence of life, e.g. amino acids, fatty acids, carbohydrates, nucleotides, aminoacyl nucleotides, oligonucleotides, phospholipids, existed on prebiotic Earth ~4 billion years ago. Substantial literature (only partly cited in the paper) exists that is dedicated to prebiotic chemistry, sources of energy, catalysts and possible environments (still not sufficiently examined) that were present on prebiotic Earth. It might be assumed that on prebiotic Earth, abiotic “metabolism” existed, through which organic substances became synthesized, activated, combined in more complex substances and structures, fell apart, and appeared again. Without such “metabolism” the emergence of life on Earth would have been impossible. The progenes could be considered as the most important specific product of abiotic “metabolism” necessary for the origin of life.

It is suggested that stable organic substances - amino acids, nucleotides (including ones of the modern day type), carbohydrates and other bifunctional agents, lipids in the form of littoral films accumulated in the ocean and washed up to the shore by high tides. Following ebb tides, organic matter concentrates through evaporation and adsorption on the ground particles, and becomes activated by sunlight (formation of activated nucleotides, dinucleotides, phosphorylated amino acids, phosphorylated sugars, phospholipids). After repeated soaking short-lived lipid vesicles are formed which *in statu nascendi* trap activated organic substances, as well as short oligonucleotides. In such lipid membranes, an admixture of highly activated phospholipids (mixed anhydrides of the phosphoric and fatty acids) might be present which could become chemical sources of energy needed for synthesis of progene components. For a short time, advantageous conditions for synthesis and combining of progenes, and therefore, for the attempts to create a bimolecular genetic system appears in lipid vesicles. As it has been described in the Consequence 1, a huge number of attempts during tens or hundreds million years result in the emergence of such system. The system occurs as a highly rare event and reproduces in the same lipid vesicle. The lipid vesicles play a role of microreactors that have necessary supply of matter and energy. After the vesicles break down, the genetic systems get into new vesicles *in statu nascendi* and continue to reproduce. Thus, they behave as virus-like organisms—protoviroids [49, 51]. In accordance with binary nomenclature, the first living organism of this kind might be named *Protoviroidum primum*, the most primitive predecessor of all living beings on the Earth. The Darvinian biological evolution “heredity—variability—natural selection” starts only after emergence of the first protoviroid.

Short characteristics of *P. primum*: a nucleoprotein, consists of single strand deoxyribopolynucleotide - gene (about 300 b, 4–5 modern nucleotides G, C, A, T, U), an admixture of ribonucleotides is possible, G + C approximately 70–75 % (but if A* was used instead of A, G + C ~50 %); a globular acidic protein with a processive polymerase (progene ligase) properties, around 100Aa, globular diameter ~ 20 Å, 10–12 canonic and non-canonic Aa (see Table 1), has hydrophobic regions for interaction with lipid membranes; the way of life—discontinuous, virus-like; the environment—abiotic lipid vesicles containing activated substances. Replicates on the template principle on the basis of progenes (RTT process) and evolve on Darwinian principle. A membrane and “metabolism” are not integral parts of the protoviroid and do not control by its genetic system.

The bimolecular genetic system is suitable for the Darwinian evolution: changes in the genome will change properties of the protein, and with positive changes, population of mutants will multiplicate (the natural selection). Microevolution will improve property of the polymerase. Macroevolution will be implemented by duplication of the first and thereafter—of successive genes [98, 99]. New genes that are useful for the system will be retained. The protoviroid will become di-, tri-, polygenic. At first, genes that are responsible for the synthesis of progenes have to emerge and become permanent (progene synthetases, amnoacyl nucleotide synthetases, dinucleotide synthetases). Arising of these genes increases effectiveness and accuracy of translation. Then genes will appear for the energy system (phosphokinases, dehydrogenases; ATP will start being used as energy currency). Proteins will appear that stabilize lipid membrane and span it for the purpose of transporting charged molecules. After the appearance of phospholipid synthesizing enzymes, a gradual transition from the protoviroid world to the protocellular one will occur. This protoviroid period is relatively short (~1–2 million years). The protocell period could last for hundredth million years. Primitive protocells will contain many genomic copies, complete and incomplete (parasitic). Molecular parasitism is an important engine of evolution, and the basis for the emergence of viruses, plasmids, transposons, and introns (51). After emergence of protocells, the cellular energetic system (glycolysis) appears. RNA will emerge, and processes of replication, translation, and transcription will become separate. Genetic function will be kept in DNA, which will become double stranded. 5′ deoxyribonucleotides, which will be synthesized from ribonucleotides (apparently more available in prebiotic conditions), will start being used for replication and 5′ ribonucleotides for transcription. Internal RNA world is appeared. It is at this stage that ribozymes are appeared, including peptidyl synthetase [30, 42, 43]; on this stage ribozymes have an important advantage over proteins in precision of their encoding. tRNA, aminoacyl tRNA synthetases and ribosomes emerge step by step; the genetic code will be gradually approaching the modern one on principle “physico-chemical group code → physico-chemical + enzymatic code → individual enzymatic code”. Developing a modern translation system will at first provide for the early (“progenic”) system of translation, and will further become independent. Evolution of translation is the main way of transition to the modern cells [37, 40, 97]. The protocellular life passes through a “bottle neck”: the last universal common ancestor (LUCA) appears, from which the modern cellular life once evolved.

In accordance with the progene hypothesis, the general scheme of biological evolution is as follows: prebiotic world (and prebiotic chemical evolution) → monogenic protoviroid (and biological Darvinian evolution) → polygenic protoviroids → polygenomic protocells → monogenomic protocells → LUCA → modern cells (or prebiotic world → protoviroid (nucleoprotein) world → protocellular DNA-RNA-protein world → LUCA → modern cell world). These schemes exclude the existence of an independent RNA world as predecessor of the cellular world.

## Conclusions

The progene hypothesis introduces concept of the first genetic system precursors—progenes forming in prebiotic environments. The progenes are amino acylated trinucleotides with amino acid specific to the first dinucleotide of the progene. They are the sole substrate for the simultaneous synthesis of the polynucleotide and polypeptide, while the sequence of amino acids gets encoded in the polynucleotides, and the emerging polypeptide possesses properties of the processive polymerase (the progene ligase). The hypothesis suggests a single mechanism that would explain: 1) arising of the genetic code; 2) mechanism of selection of constituents of genetic system, including arising of homochirality; and 3) emergencе and reproduction of a bimolecular genetic system consisting of a polynucleotide gene and a protein processive polymerase encoded in that gene. The nature, the way of existence, and the evolution of the first living organism *Protoviroidum primum* are briefly discussed.

Testing of some of the elements of this hypothesis involving stereochemical analysis and chemical experiments on synthesis of progenes is principally possible and has to help in creation of a new concept of the origin of life instead of the RNA world idea, unabled to explain the origin and nature of the first living being. I suppose that the primary RNA world never existed and ribozymes arouse on early stage of the protocell world as important addition to primary protein enzymes.

## Reviewers’ comments

### Reviewer’s report 1: Dr. Thomas Dandekar

The article is from an exciting field of speculation and theory, the origin of life. The nucleoprotein world is an interesting concept, stressing the tight connection between first nucleoproteins and first peptides giving rise to a primitive nucleic acid and polymerase system. The record of earlier proponents of this theory is correctly given. Some figures illustrate the theory. Like often in this field and only human, the author is convinced of his theory and fails in some respect to appropriately cite and consider alternative hypotheses, cross links to other (even noble price winning) much earlier work or (admittedly most challenging but the real work) meticulously collect observable evidence for his theory favoring it over alternatives.

Dear author, according to my recommendation and opinion your theory should be given more in context. With current knowledge, each of any of the exciting “origin of life” theories stresses one aspect of the origin of life, in your case the coevolution of nucleic acids and proteins while almost necessarily neglecting others. Specifically (major—3 major points really necessary to add): 1—Cite and explain at least the following three major alternative theories in addition to your otherwise good background (in particular you mention the otherwise forgotten Russian pre runners, good work!): Theory of surface metabolism (Wächtershäuser as a main proponent), as surfaces and selection of structure work even in absence of genetic code or nucleoproteins, so a plausible pre-runner to your theory Theory of Hypercycle evolution by Manfred Eigen (nobel prize winner) giving even quantitative details on coevolution of molecular species. Furthermore, it explicitly covered coevolution of nucleic acids and proteins. Theory of membrane evolution (e.g. work by Gareth Griffith), again the conundrum is the evolution of structures if there is no genetic code around. An alternative solution is that membranes pass on their structure and serve in this way as replicating templates without genes. 2—Once you have done this, you can better appreciate the limitations and strengths of your well taken important aspect in the early evolution of life and place your theory in the context of more general approaches as those cited above. 3—To be even more useful (of course such ideas are always stimulating, help to progress in fundamental science and understanding of evolution as well as basic cellular processes) you should furthermore collect some more observable evidence for your theory and give that in a Table, or if more and substantial, as figures. The three approaches cited above have done quite well in this respect and help you to see what could be added regarding experimental evidence and direct observations.

Author’s response: *Thank you very much for your criticism and remarks*, *for wishing to make better my paper. The goal of this paper was not to do a complete review of different theories of the origin of life. I included about 20 additional citations in the paper in accordance with your recommendations and advises of other reviewers*.

## Reviewers’ comments

### Reviewer’s report 1/2

Dear Author, thank you for your revision. Please follow my original comments and revise point-by-point your manuscript. I do not change a major shift in the present version compared to previous. The start of life is a fascinating topic. Your theory covers one aspect, the Nucleoprotein World explains how a link between peptides and nucleotides could be established. However, this is only one explanation, it is also not that novel and it should be explained in context. In particular, you should not give a statement such as “how life began” as you are not covering earlier stages such as surface metabolism or origin of membranes but more the step of development towards first genetic codes. For instance this feature should be presented in the proper context and it does not take away anything from your creative idea and even strengthens the presentation. Furthermore, if you should have any type of data supporting your theory this is another plus. Only if these changes are fully incorporated I would think your idea is appropriately presented and I can endorse the manuscript.

Author’s response: *I added the text on Manfred Eigen’s “Hypercycle” on p.2. Indeed it is important for discussion on the genetic system arising. I also included an additional data on the surface metabolism and hydrothermal vents into the last section (p. 9–10). A short discussion is also presented on primitive membranes. I think it is not main point of the problem. On my opinion, the main and most complicated question of the problem “how life began” is the molecular mechanism of emergence of a bimolecular genetic system.*

## Reviewers’ comments

### Reviewer’s report 2: Dr. Bojan Zagrovic

The article by Altstein reviews a body of mostly theoretical work aimed at postulating a coherent framework for explaining the origin of the first bimolecular genetic systems involving nucleic acids and polypeptides. The central concept behind this framework is that of a progene—an aminoacylated trinucleotide in which the 3′–attached amino acid is in some way physicochemically specific for the 5′ dinucleotide of the progene. Progenes, then, are substrates for the simultaneous processes of nucleic acid replication and polypeptide translation. Importantly, in this scenario, the encoded polypeptide is a polymerase involved in the progene ligation reaction. The strengths of the article are, in my opinion, threefold. First, as a welcome challenge to the RNA world hypothesis, the idea that the worlds of nucleic acids and polypeptides were chemically, functionally and evolutionarily intertwined from the very start presents a host of falsifiable, testable hypotheses, which could and should be taken into the account and directly tested. For example, recent results by the Sutherland group showing that key RNA, protein and lipid precursors can indeed be simultaneously obtained using cyanosulfidic chemistry under prebiotic conditions (Patel et al. Nature Chemistry, 7, 301–307, 2015) suggest that the origin-of-life scenario involving a coevolution of the three groups of biomolecules from the very beginning should be seriously considered. The second strength of the article and the progene idea itself is that it within one self-consistent framework proposes an explanation for: 1. the origin of the genetic code, 2. a mechanism for the selection of the elements of the genetic system and, finally, 3. the appearance of a simple genetic system involving a gene and a gene product. It is my belief that indeed any reasonable framework in this context must have the ability to account for these different aspects of living systems and their origin simultaneously. Finally, a relevant contribution of the article is that it summarizes and brings to the English-speaking audience some of the old literature on the topic, mostly by the author himself, which originally appeared in the 1980s only in Russian. Major comments 1. An important weakness of the progene proposal, as also acknowledged by the author, is that the selection rules which would link the appropriate amino acids and the corresponding trinucleotides are still largely unclear and vague from the physicochemical point of view. The author mentions the results of some modeling efforts in this direction, but these still appear to be largely preliminary. If these preferences were indeed used for the establishment of robust replicating and translating systems, it is my expectation that they indeed should be quite robust and detectable themselves. Our own recent efforts in the direction of decoding the intrinsic preferences of amino acids and nucleotides and linking them with the structure of the genetic code might in part serve this purpose (Hlevnjak et al. NAR, 40, 8874–8882, 2012; Polyansky et al. NAR, 41, 8434–8443, 2013 and de Ruiter et al. NAR, 43, 708–718, 2015). The article would be improved by including a more extensive discussion of such or similar details about what is already known about amino-acid/nucleobase preferences. 2. Second the article would be more balanced if the author discussed other similar proposals in the direction of simultaneous and interlinked evolution of nucleic acid and polypeptide systems. In particular, the work by Charles Carter and coworkers (reviewed, for example, in Life, 5(1), 294–320, 2015) and John Sutherland and coworkers comes to mind in this context. In particular, the latter has shown that aminoacylated trinucleotides can form abiotically and has proposed that selective interaction within such complexes might have served as the basis for the development of genetic encoding, in a similar manner as proposed herein (Borsenberger et al. Chem. Biodivers 1, 203–46, 2004 and Biron et al. Angew. Chem. Int. Edit, 44, 6731–6734, 2005). 3. Finally, concerning the progene forming mechanism, which is the very core of the proposed model, the author discusses the lifetimes of unstable triplets as the key element determining their suitability for overlapping with other complementary triplets. On the other hand, even if the lifetimes are long enough, but the on rates are too slow, the proper complexes will have a difficult time forming. In the end, what matters are both the on and the off rates. This is not a strong criticism considering that the details of the progene idea are anyways still underdeveloped at this level—the main strength of the article is in any case in presenting a general, testable framework with a number of individual details like this still to be fully developed and appropriately tested.

Author’s response: *Thank you very much for carefully reading of my paper and giving a positive estimation of the progene hypothesis. I agree with your remarks on the hypothesis. Our stereochemical analysis shows that the postulated mechanism of the progene formation is apparently possible: we found that length of a 3′ “tail” of the progene is enough for an interaction between an amino acid of an aminoacyl nucleotide and a dinucleotide, and a specificity of the interaction between an amino acid and a dinucleotide could exist. We performed it in 1988 (ref. 50) and the data were inspiring. Modern stereochemical research would be useful of course. It is necessary a hard experimental work with chemical synthesis of postulated aminoacyl nucleotides and 3′activated dinucleotides to determine the specific role of an amino acid during the progene synthesis. The methods, developed by L. Orgel and coworker on a non-enzymatic synthesis of oligonucleotides, could be useful. On your third remark, I assume that the lifetime of unstable triplets (Fig.**1**) has crucial significance for selection of suitable components, including mutual specificity of amino acids and dinucleotides. Progene formation would be impossible, if the lifetime of imperfect ‘triplets” is shorter than time of complementary interaction between them. The rate of complementary interaction of two instable triplets (with specific amino acids) has to be approximately correspond with their lifetime. On your recommendation I included some additional references in the text.*

## Reviewers’ comments

### Reviewer’s report 3: Dr. Anthony Poole

In this speculative article, Altstein argues for the simultaneous emergence of genes, transcripts,a protein-based replicase enzyme and translation. This is a theoretical model, which was first published in 1987 in Russian in the journal Molekularnaya biologia (Ref [49]). A brief 2-page summary appeared in English in 1996 as a conference contribution in the journal Origins of Life and Evolution of the Biosphere [1]. The current version has been updated, citing a number of more recent pieces of work, so this is not just a translation of the 1987 piece, though the abstract from OLEB in 1996 does suggest the core ideas were already published. Personally, I think there is merit in publishing works such as this, that have not been as widely read as they might, owing to their not being published in English. In that regard, readers should make up their own mind as to what they think of this model. From my perspective, the challenge for a paper such as this is whether it is essentially a historical piece, or whether it may lead to new experimental study on the origin of life. I suspect there is scope for the latter, though the experiments may be tricky.

While I thought the basic model was intriguing, I found the second half of the paper to be lacking, from the section, ‘Consequence 3. The molecular mechanism of genetic code arising’ through to the end. The discussion of the genetic code, and the two sections after that seemed to have been written with little consideration of the literature on the genetic code, prebiotic chemistry or the evolutionary transitions proposed leading to modern cells. There were few references, the explanations were a bit dense in places. For instance, the section on the genetic code ends with a statement claiming that the progene hypothesis can explain around 8 features of the code (note the list is misnumbered), but the preceding text is not really sufficient to allow the reader to assess such a bold statement. I was also surprised to see in that section a brief mention of a preliminary analysis (p7, line 30), which appears to date back to a conference abstract from 1996. Surely the author has had occasion to complete such work by now! The next two sections appear to be a summary of the author’s opinion on the evolution of cells from the starting point of progenes. Again, this is poorly cited, and largely ignores the literature. Overall, the last three sections are a bit weak to be published in their current form. In updating the paper to reflect subsequent research, there are several matters that might helpfully be discussed: − Nucleotide cofactors—for instance S-adenosylmethionine—how are these different (or similar) to the aminoacyl nucleotides proposed?—Several authors have proposed tRNAs as cofactors (e.g. Szathmary [2]) and a relationship between codons and amino acid binding capacity (e.g. Yarus [3])—how are they different from the system proposed here?—Recent work shows that current in vitro ribozymes are able to copy sequences that are as long as themselves [4], indicating that, at least chemically, that issue with the RNA world model is not a major one. It would seem appropriate to discuss this development.—A number of others have argued that an RNA world model is problematic, and that translation may have had chemical origins. A recent paper by Bowman et al. [5] is perhaps the most comprehensive on this topic, and warrants mention, if only to note that others are also discussing the plausibility of a nucleoprotein world. Finally, the figure legends are too brief to be helpful, and the figures in the addendum do not help the reader in understanding the discussion of the dinucleotides on pp7–8. References [1] Altstein AD (1996) The origin of protocells. Origins of Life and Evolution of the Biosphere 26(3–5):477–8. [2] Szathmary E (1999) The origin of the genetic code: amino acids as cofactors in an RNA world. Trends Genet 15(6):223–9 [3]. Yarus M, Caporaso JG, Knight R (2005) Origins of the genetic code: the escaped triplet theory. Annu Rev Biochem. 74:179–98 [4]. Attwater J, Wochner A, Holliger P (2013) In-ice evolution of RNA polymerase ribozyme activity. Nature Chemistry 5:1011–18 [5]. Bowman JC, Hud NV, Williams LD (2015) The Ribosome Challenge to the RNA World. Journal of Molecular Evolution 80(3–4):143–161.

Author’s response: *Thank you very much for reviewing my paper and for your remarks and advices. I think that your remarks are fair. However, I would like to respond to some of them.*

1. *A detailed analysis of the genetic code problem was not of my paper task. I only showed that the postulated mechanism of progene formation allows to explain emergence of the prebiotic genetic code by an especial physicochemical way. It is an important part of the progene hypothesis. In accordance with your and other reviewers advises, I added some references into this section and corrected the end of the section. The references 39, 40, 76, 77 contain comprehensive reviews of the problem. Our stereochemical analysis had produced some encouraging data but they are need to be tested much more extensively and with new edition of the progene 3′ “tail”. I combined two last sections, in which I aim to show my opinion on the origin of cells on the basis of the progene hypothesis but don’t aim to disscus the origin of cells in general. I added some references into this section.*
2. *I hope that the figures will be more understandable if readers use the legends as well as the text of the corresponding sections. I would not like to drop the Addendum because it may be useful for readers who familiar the molecular models of nucleotides.*
3. *I would like to add an important remark on ribozyme polymerases. It’s known that there are two types of polymerases: distributive (they can make phosphodiesther bond between two nucleotides but can’t move along a template; many molecules of such a polymerase are needed to copy a template) and processive (they bind with a template, move on it and copy it; one molecule of the polymerase is enough to copy the template). Only a processive polymerase is significant for the origin of the first genetic system because a number of molecules with polymerase properties can’t arise simultaneously. There is no evidence at present for existence any processive ribozymes (natural or artificial). All of them are distributive ones. Ribosomes and telomerases containing ribozyme parts can’t move on a template without their protein parts. Apparently short polynucleotides (<200 bases) can’t be processive polymerases but long ones can’t arise in racemic milieu due to the stereochemical inhibition phenomenon and other obstacles (see references* [31–33]*). I think, this is a big challenge for the RNA world hypothesis.*

## Abbreviations

DNA: Deoxyribonucleic acid
RNA: Ribonucleic acid
PDE bond: Phosphodiesther bond
AAN: Aminoacyl nucleotide
Aa: Amino acid
DN: Dinucleotide
LUCA: Last universal common ancestor
ATP: Adenosine triphosphate
D, L: D- and L-stereoisomers of nucleotides and amino acids
RTT: Replicative transcription-translation
A*: Diaminopurine nucleotide
T, U, C, A, G: Standard nucleotides
